# Metabolomic Approaches in Fermented Meat Products: Focus on Lactic Acid Bacteria and Starter Cultures

**DOI:** 10.3390/microorganisms14071591

**Published:** 2026-07-21

**Authors:** Marianthi Sidira, Grigorios Nelios, Theodoros Varzakas

**Affiliations:** 1 Laboratory of Microbiology, Biotechnology & Hygiene, Faculty of Agricultural Development, Democritus University of Thrace, 68200 Orestiada, Greece; sidiramania@yahoo.gr; 2 Laboratory of Applied Microbiology and Biotechnology, Department of Molecular Biology & Genetics, Democritus University of Thrace, 68100 Alexandroupolis, Greece; gregnelios@hotmail.com; 3 Department Food Science and Technology, University of the Peloponnese, 24100 Kalamata, Greece

**Keywords:** metabolomics, lactic acid bacteria, fermented meat products, starter cultures, GC-MS, LC-MS, NMR, volatile compounds, biogenic amines

## Abstract

Lactic Acid Bacteria (LAB) and other starter cultures play key roles in fermented meat products by influencing fermentation, safety, sensory properties, and metabolite formation. Metabolomic approaches based mainly on mass spectrometry, including GC-MS, LC-MS, and CE-MS, as well as nuclear magnetic resonance (NMR), provide useful tools for characterizing volatile and non-volatile metabolites, monitoring quality, and identifying candidate biomarkers. This narrative review summarizes recent studies from the past seven years on metabolomic approaches applied to fermented meat products, with emphasis on LAB, starter cultures, and metabolites related to quality, safety, and fermentation. Overall, metabolomics can support the holistic characterization of fermented meat products and improve understanding of microbial activity during fermentation and ripening.

## 1. Introduction

The Lactic Acid Bacteria (LAB) comprise a diverse group of Gram-positive bacteria, catalase-negative, acid tolerant, non-respiring non-spore-forming, cocci, or rods with low guanine + cytosine (G + C) content. Their main metabolic feature is the production of lactic acid as the principal end product of carbohydrate fermentation [1,2]. According to Mozzi [2], LAB are generally regarded as nonpathogenic microorganisms with Generally Recognized as Safe (GRAS) or food-grade status; however, exceptions exist among species belonging to the genera Streptococcus, Enterococcus, Lactococcus, and Carnobacterium. Therefore, safety should not be generalized to the entire LAB group but should be considered in relation to the specific species and, where relevant, strain. As a result, LAB are used in a wide range of foods such as dairy products including fermented milk, yoghurt, or Feta-type cheese [3,4,5,6,7], fermented fruits, fermented vegetables, and vegetable juices [8,9,10], meat products for example dry-fermented sausages [11,12,13,14,15], or even sour meat [16] and wine [17].

Specific strains may be considered probiotic only when their health benefits for the host have been demonstrated by appropriate studies [18]. Additionally, in fermented meat products, some LAB strains are well adapted to meat environments [19] and can influence the profile of volatile compounds [20].

**Figure 1:**
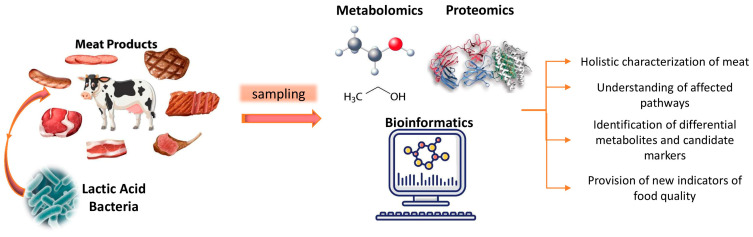
Schematic representation of omics technologies, such as metabolomics and proteomics, in meat products.

It should be noted that LAB can produce a variety of metabolites, like organic acids, bacteriocin, amino acids, exopolysaccharides, and vitamins. For this reason, the balance as well as the stability of gut microbiota can be maintained [21]. Considering this and the complexity of the food matrix, omic technologies (metabolomics, proteomics etc.) alone or in combination can be used for the holistic characterization of meat products inoculated with LAB, firstly, to understand the mechanisms and affected pathways in meat quality; secondly, to identify differential metabolites and candidate markers; and thirdly, to provide new indicators of food quality like organoleptic characteristics, genetic origin, etc. (Figure 1).

A metabolomic approach of LAB can also be used in order to compare the strains origin through monitoring the consumption of amino acid and accumulation of organic acids as well as volatile organic compounds [19]. Chemical changes caused by the presence of LAB and the metabolic output of meat product. Therefore, metabolome profiling together with multivariate analysis is a useful approach to distinguish naturally and artificially inoculated samples [22].

**Figure 2:**
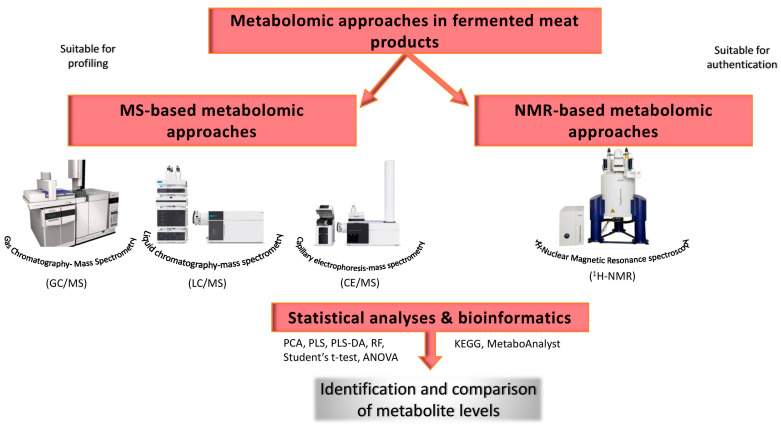
Metabolomic approaches applied to fermented meat products.

Generally, two main analytical platforms have been applied in metabolomic studies (Figure 2): mass spectrometry (MS)-based approaches and nuclear magnetic resonance (NMR)-based approaches. In MS-based metabolomics, separation is usually achieved by chromatographic or electrophoretic techniques, such as GC, LC, or CE, followed by MS detection. By contrast, NMR-based metabolomics is a non-destructive approach that can provide metabolic fingerprints with minimal sample preparation [23]. MS-based approaches offer higher sensitivity and broader metabolite coverage [24,25,26], whereas NMR is rapid and reproducible but has lower sensitivity and may suffer from spectral overlap [27]. Both analytical platforms can be combined with statistical and bioinformatics tools to identify biomarkers for food quality monitoring and to explore molecular pathways related to key metabolites [22].

The aim of the present review is to summarize metabolomic approaches applied to fermented meat products, with emphasis on lactic acid bacteria, starter cultures, and metabolites related to quality, safety, and fermentation. Our search was carried out in Scopus and used the following combination of keywords: metabolomics and meat; metabolomics and LAB; metabolomics and meat and LAB. Here, we summarize the latest studies regarding the application of advanced omics technologies in LAB and meat products.

## 2. Omics Technologies in LAB

LAB are used in a wide range of fermented foods. During metabolism they can produce a variety of metabolites, like short-chain fatty acids, amines, bacteriocins, exopolysaccharides, and vitamins [28]. Some of the LAB are highly adapted to meat environments [19] and have also been affected the composition of volatile compounds in meat products [20] by affecting the level of metabolites during fermentation [29]. Thus, metabolomic approach of LAB can be used in order to compare the strains origin through monitoring the consumption of amino acid and accumulation of organic acids as well as volatile organic compounds [19]. The cause of the improvement of physicochemical characteristics of fermented meat products is the nitrate/nitrite degradation, antibacterial and antioxidant metabolites, and lactic acid bacteriocins of LAB during the fermentation process [30,31]. Meanwhile, LAB are used to increase the nutrition of food; mitigate the level of harmful microorganisms; prolong shelf-life; improve the color, flavor, and pH of meat products; etc. [28,32].

According to Wang et al. [29], 29 major differential metabolites together with 23 volatile compounds and the top 10 bacteria were selected from low-salt meats in order to be subjected to correlation analysis. According to the results, *Lactobacillus* and *Pediococcus* were significantly correlated with aldehydes, acids, alcohols, and the majority of the esters. Both of them can breakdown carbohydrates through lactate dehydrogenase and generate lactic acid which is a precursor substance for 3-hydroxy-2-butanone. Furthermore, *Lactobacillus* and *Pediococcus* were positively correlated with linolenic and linoleic acid, which were also correlated with volatile flavor compounds, for example hexanal, nonanal, heptanal, and 1-octen-3-ol. This is because of lipid degradation, which functions as the key source of volatile compounds during product fermentation. Apart from this, Lactobacillus, *Lactococcus*, and *Pediococcus* were positively correlated with free amino acids like valine, leucine, and phenyl-alanine, which strongly affected the overall flavor of fermented meat.

Moreover, metabolomic analysis of *Pediococcus pentosaceus* isolated from Harbin dry sausages was performed [33]. To clarify the antioxidant response at the metabolic level, changes in the metabolic profile under oxidative stress were assessed using GC-TOF-MS-based metabolomic analysis. In total, 326 metabolites were detected: 141 were known and 15 were unknown metabolites. More precisely, for the 141 known metabolites, amino acids had greater percentage of individual components succeeded by organic acids, carbohydrates, nucleotides, fatty acids, lipids, alkylamines etc. In total, 74 differentiated metabolites were classified into eleven categories. Of these, 27 amino acids were the most altered metabolites, followed by 15 nucleotides, nine carbohydrates, nine organic acids, nine lipids, and three fatty acids.

*Staphylococcus* was the dominant genus during the resting and ripening of Panxian ham fostering the production of amino acids and fatty acids [34]. Due to its strong enzyme activity, including nitrate reductase, catalase, lipolytic, and proteolytic enzymes, coagulase-negative staphylococci is a key factor in the flavor and taste development of Panxian ham. Further, the respective Staphylococci contributed to the extension of product shelf life through nitric oxide synthase, showing strong antagonistic behavior against undesirable microorganisms. Pathogenic microorganisms were reduced and finally disappeared during traditional spontaneous fermentation.

Fuet fermented sausages, a traditional Spanish dry-fermented sausage [35,36], have been studied by Yang et al. [37] and compared to inoculated fermented sausages of different species. UHPLC-QTOF-MS-based metabolomic profiling revealed that commercial starter cultures were unable to reproduce the metabolomic profile of traditional sausages, mainly due to the limited diversity of Staphylococcus species. However, the study demonstrated variation in metabolites within inoculated fermented sausages, indicating that microbial variation of commercial starter cultures modified the metabolic profile of sausages [37]. As revealed by metabolomic analyses, several coagulase-negative *Staphylococcus* species (such as *S. carnosus*, *S. xylosus*, *S. equorum, S. saprophyticus*) were closely associated with the production of esters, methyl aldehydes, and ketones in Fuet fermented sausages without starter culture, while only *S. carnosus*, *S. xylosus*, or *S. xylosus* were positively correlated with the production of aldehydes, ketones, and esters or acids, alcohols, and ketones, respectively, in inoculated fermented sausages with commercial starter cultures.

According to Zhu et al. [38], *Staphylococcus* was the dominant non-LAB bacterial genus in dry-cured ham of five different origins (China: Jinhua, Xuanwei, Rugao; Spain: Iberian; and Italy: Parma) and was associated with the formation of quality-related metabolites. Microbial diversity was assessed by 16S rRNA sequencing, whereas microbial and metabolite biomarkers were characterized using LC-Q-TOF-MS-based metabolomic analysis in the dry-cured ham samples. This genus was positively correlated with amino acids and lipid metabolites in dry-cured ham, such as ethyl tetradecanoate, ricinoleic acid, inosinic acid, sciadonic acid, taurine, linoleic acid, oleic acid, pyrophosphate, and carnosine. These findings suggest that *Staphylococcus* may contribute to the production of amino acids and fatty acids during dry-cured ham ripening.

In a recent study on reduced-nitrite Chinese fermented sausages containing gallic acid, the non-LAB genus *Staphylococcus* was positively correlated with dimethylamine and trimethylamines well as specific peptides and free amino acids, whereas *Lactococcus* species (*L. garvieae*, *L. formosensis*, and *L. lactis*) showed a negative correlation with these compounds. Gallic acid regulated amino acid metabolism by inhibiting the spoilage bacteria, while it increased the growth of LAB [39].

The impact of *L. sakei*, *P. pentosaceus*, and *S. xylosus* on the metabolomic profile of Italian salami (a type of dry-fermented sausage) was evaluated by Rocchetti et al. [40]. According to that study, *S. xylosus*, *P. pentosaceus*, and especially *L. sakei* revealed an optimal adaptation to the meat matrix, and LAB was the dominant microbial group during the whole ripening period of the Italian salami samples, confirming their suitability as starter cultures accordingly. Nevertheless, these starter cultures presented diverse chemical profiles, with *L. sakei* mainly associated with lipid- and protein-related changes and *P. pentosaceus* with increased fatty acyls, organonitrogen compounds, and remarkably, γ-glutamyl peptides. Certainly, *L. sakei* played the major role in changing the metabolomic profile of Italian salami during ripening. Overall, these findings indicate that the formation of metabolomic profiles is partly driven by microbial activity and partly associated with physicochemical and biochemical changes occurring during ripening. Mixed starter cultures containing *L. sakei*, *P. pentosaceus*, and *S. xylosus* in Italian salami were also studied. Using UHPLC-HRMS-based metabolomic analysis, 144 discriminant metabolites were recorded, and arginylserine was detected as an exclusive metabolite. The above mixed cultures deliver a great enhancement of amino acid and peptide levels, followed by those of pyrimidines, purines, and imidazoles, owing to the fact that complex interaction of numerous metabolic activities by the different strains take place in the dry-fermented sausages during the ripening process.

Finally, the impact of *Pediococcus acidilactici* on the metabolomic profile of fermented dry-cure mutton sausages was evaluated by Jiang et al. [41]. According to this study, the addition of *P. acidilactici* improved the flavor of sausages and was highly correlated with a variety of metabolites like esters and aldehydes (positively correlated). Additionally, with regard to the inoculated sausage fermentation associated with the formation of amino acids and peptides, it was observed that after inoculation, the levels of eight amino acids and four peptides were significantly increased. In the current study, *Pediococcus* promotes the syntheses of Val, Pro, Orn, as well as the formation of secondary alcohols such as phenethyl alcohol and 1-octen-3-ol. It is important to note that the addition of starter culture increased the overall abundance of LAB and decreased the number of spoilage bacteria.

MS-based metabolomic technique was also used to understand the growth characteristics of *Lactobacillus* and associated energy production by means of lactic acid fermentation. More particularly, sugars, organic acids, amino acids, and adenosine derivatives were significantly changed during cell growth and 10 cell metabolites (carnitine, triose phosphate, 5-deoxy methylthioadenosine, phosphoric acid, phenylalanine, citric acid, talose, lysine, inositol, and trehalose) together with adenosine derivative (5-doexy methlylthioadenosine) were identified as major metabolites [42]. Metabolomic approaches applied to LAB and other starter cultures in fermented meat products have been reported in Table 1.

**Table 1:** Metabolomic approaches applied to LAB and other starter cultures in fermented meat products.

Microorganism/Starter Culture	Extraction Method	Metabolite Identification Methods	Data Analysis	Results	References
*Pediococcus pentosaceus*	hot ethanol method	GC-TOF-MS	PCA, OPLS-DA, SPSS 25.0, ANOVA	In total, 326 metabolites were detected: 141 were known and 15 unknown; 74 differentiated metabolites were classified into eleven categories, primarily amino acids and nucleotides.	[33]
*Staphylococcus* spp.	V_Methanol_: V_Chloroform_ = 3:1), then 5 mL of L-2-Chlorophenylalanine (1 mg/mL stock in dH_2_O) was added as internal standard	GC-TOF-MS	RDA triplot analysis, KEGG	Significantly correlated with changes in differentiated metabolites during Panxian ham processing. Production of all amino acids and fatty acids except stearic acid.	[34]
*Staphylococcus* (*S. carnosus, S. xylosus, S. equorum, S. saprophyticus*)	headspace solid-phase microextraction	UHPLC- QTOF-MS	PCA, PLS-DA, Pearson’s correlation coefficient (PCC)	Closely associated with the production of esters, methyl aldehydes, and ketones in Fuet fermented sausages.	[37]
*Staphylococcus* spp., *Tetragenococcus, Halomonas*	V methyl butyl ether: V methanol = 5:1),	LC-Q-TOF-MS	Student’s *t*-test, PCA, OPLS, one-way ANOVA	Positively correlated with amino acids and lipid metabolites, ethyl tetradecanoate, ricinoleic acid, inosinic acid, sciadonic acid, taurine; linoleic acid, oleic acid, pyrophosphate, and carnosine in dry-cured ham.	[38]
*Staphylococcus saprophyticus.*	QuEChERS method (Quick, Easy, Cheap, Effective, Rug-ged, and Safe) solid phase extraction	UHPLC-MS/MS	ANOVA, PCA, OPLS-DA	Positively correlated with dimethylamine and trimethylamines well as specific peptides and free amino acids in reduced-nitrite Chinese fermented sausages.	[39]
*Lactococcus (L. garvieae, L. formosensis, L. lactis)* *Macrococcus*	QuEChERS method (Quick, Easy, Cheap, Effective, Rugged, and Safe) solid phase ex-traction	UHPLC-MS/MS	ANOVA, PCA, OPLS-DA	Negative correlated with dimethylamine and trimethylamines as well as specific peptides and free amino acids in reduced-nitrite Chinese fermented sausages. Gallic acid (GA) promoted the growth of *Lactococcus* while suppressing the proliferation of spoilage bacteria and *Macrococcus.*	[39]
*Latilactobacillus sakei, Pediococcus pentosaceus, Staphylococcus xylosus*	homogenizer-assisted ex-traction method	UHPLC-HRMS	HCA, PCA, OPLS-DA	Less sensitive to lower pH values. Major role in modifying metabolomic profile during ripening. Activity involving lipids and proteins in Italian Salami, dry-fermented sausages. Proteolytic activity. When mixed with *Latilactobacillus sakei* and *Pediococcus pentosaceus* for the production of Italian Salami, 144 discriminant metabolites recorded. Arginylserine was the exclusive metabolite. The above mixed cultures deliver a great enhancement of amino acid and peptide levels, followed by those of pyrimidines, purines, and imidazoles.	[40]
*Pediococcus acidilactici, Rhizopus oryzae*	SPME (7.5 mL of sodium chloride and 15 μL of the standard in-ternal Cyclo-hexanone)	UHPLC-MS/MS	ANOVA, PCA, PLS-DA,	Highly correlated with a variety of metabolites like esters and aldehydes (positively correlated). Associated with the formation of amino acids, and peptides. Eight amino acid and four peptide contents were significantly increased after inoculation. Promote the formation of secondary alcohols (phenethyl alcohol and 1-octen-3-ol). Improve the flavor of fermented dry-cure mutton sausages.	[41]

Hierarchical Cluster Analysis (HCA), Principal Component Analysis (PCA), Orthogonal Projections to Latent Structures Discriminant Analysis (OPLS-DA), Partial Least Square Discriminant Analysis (PLS-DA), Analysis of variance (ANOVA), Honestly Significant Difference (HSD), Pearson Coefficient Correlation (PCC), Statistical Package for the Social Sciences (SPSS), Kyoto Encyclopedia of Genes and Genomes (KEGG).

## 3. Omics Technologies in Meat Products or Cured Meat Products Inoculated with LAB

Traditional dry-cured meat products produced by fermentation and ripening are based on microbial and enzymatic activities for flavor formation [43,44,45,46]. Microbial communities responsible for the ripening of meat product were investigated [37,47,48,49] giving a microbiota viewpoint of the respective products. In general, it seems that microbial species diversity could lead to various metabolic behaviors that possibly influence the volatile and nonvolatile metabolite profiles of the fermented meat products [37]. This can be seen in Reckem et al. [50], where the use of commercial starter cultures in meat products, including *Pediococcus pentosaceus* and *S. xylosus*, produces a mild and more “Mediterranean” flavor, or in Ferrocino et al. [49], where the use of *Latilactobacillus sakei*, *Latilactobacillus curvatus*, and the coagulase-negative staphylococcus species *S. xylosus* affected the volatilome profile of fermented sausages.

Apart from fermented sausages, chicken meat products or generally ready to eat meat products have been associated with several foodborne pathogens or spoilage bacteria that can affect shelf-life and food safety [51,52]. In addition to this, significant metabolite changes occurred at the end of ripening. Therefore, metabolomic analyses have been used extensively for the assessment of meat quality as well as to investigate the quality traits of meat products [53,54].

More specifically, Rocchetti et al. [54] investigated the metabolomic profile of nitrate-free salami during ripening using UHPLC-QTOF-MS-based metabolomic analysis and identified 111 metabolites. Of these, fatty acyls (31 compounds) and glycerol phospholipids (27 compounds) were the main ones, followed by prenol lipids (10 compounds) and other metabolites like steroids and hydroxyl-fatty acids. Taking into consideration the findings, the cold drying ripening process could be a great way for the production of dry-fermented sausages without additives (nitrates/nitrites).

Sour meat, a traditional Chinese fermented meat product, was recently investigated by Wang et al. [29]. According to this study, GC-MS was used to examine metabolite changes during low-salt fermentation. A total of 68 and 77 differential metabolites were identified in low-salt and traditional sour meat, respectively, including organic acids, amino acids, peptide amides, ketones, aldehydes, sugars, lipids, nucleic acids, and their derivatives. The level of free amino acids in low-salt sour meat was higher than that in traditional sour meat at the end of fermentation, suggesting that protein hydrolysis can be promoted by salt reduction in fermented meat products. Moreover, due to the low-salt concentration, the maturation of meat products can be accelerated, enhancing their nutritional value. The authors also suggested that the increase in free amino acids was associated with the richer microbial diversity observed during low-salt fermentation.

In addition to this, ^1^H NMR technique was applied to characterize the bioactive metabolites linked to the antibacterial activity of chicken meat marinated with fermented ginger paste. In that study, 25 metabolites were detected in the fermented ginger paste, including 13 aliphatic compounds, 10 sugars, and two aromatic compounds. It is worth noting that the concentration of sucrose was lower than that of glucose and fructose because of its utilization during fermentation. Consequently, more monosaccharides like glucose and fructose were generated [55].

To evaluate metabolite differences among Fuet sausages, including spontaneously fermented sausage and sausages inoculated with two different commercial starter culture combinations (*P. pentosaceus*, *P. acidilactici*, *S. xylosus* and *S. carnosus*; or *P. pentosaceus* and *S. xylosus*), UHPLC-QTOF-MS-based metabolomic profiling was performed. In total, 142, 149, and 120 differential metabolites were identified, respectively, including amino acids, dipeptides, carbohydrates, organic acids, fatty acids, and phosphocholine derivatives. The metabolomic profiles of these three fermented sausages were significantly different. The use of starter cultures was positively associated with amino acids, fatty acids, L-anserine and L-carnosine levels, suggesting that inoculation can increase amino acid and fatty acid content and contribute to dipeptide formation [37].

GC-TOF-MS-based metabolomic analysis was used to investigate the relationship between microbial communities and metabolite formation during the spontaneous fermentation of Panxian Ham, a traditional Chinese dry-cured ham. In total, 226 metabolites were detected and 31 significantly different metabolites were identified including 15 amino acids, six fatty acids, three organic acids, two sugars, two polyols, two nucleic acids, and one additional metabolite [34].

Sugimoto et al. [45] used CE-MS-based metabolomic analysis to quantify several hydrophilic molecules in dry-cured ham, including amino acids (glutamine, cysteine, asparagine and leucine), organic acids, peptides, nucleotides, and related intermediate metabolites. Amino acid, nucleotides, and organic acids concentration were gradually increased during ripening, resulting an improvement in sensory evaluation. Moreover, ripening time could be reduced from 680 to 540 days without affecting the product flavor and quality.

Black pig dry-cured ham was investigated by Shi et al. [46] using the SPME GC-MS technique. That study identified 407 volatile compounds in total during the ripening process, with the main volatile compounds formed at 210 days of ripening. Aldehydes and alcohols were the most abundant flavor compounds, originating from fatty acids oxidation as well as amino acid degradation of the meat.

NMR technique (high-resolution magic angle spinning, ^1^H HR-MAS NMR) was used in order to study the metabolomic profile of salchichón, a traditional dry-fermented sausage, during the ripening process. This non-MS based technique showed changes in metabolome profile because of the fermentation and ripening process, indicating that the NMR technique is effective for monitoring the fermentation process and classifying samples based on their ripening time. In total, 177 metabolites were identified, including amino acids, peptides and analogues, carbohydrates, organic acids and derivatives, nucleosides, nucleotides and analogues, fatty acids, and miscellaneous, such as Acetone, Crn, Cho, PCho, GPCho, or IMP [56]. This finding surmised that NMR metabolomics can monitor microbial activity compared to proteolysis and lipolysis [22].

According to Muroya et al. [22], skeletal muscle metabolites, like sugars and amino acids, are affected by animal genetic background, feeding, muscle type, postmortem aging, and meat processing. The respective metabolomic changes are associated with meat quality traits such as color, water-holding capacity (WHC), pH decline, flavor, and palatability. Regarding meat color, the redness and discoloration are affected by myoglobin’s chemical status, whereas for the WHC, by myofibrillar’s protein denaturation, resulting WHC reduction.

Zhu et al. [38] combined high-throughput 16S rRNA gene sequencing with UHPLC-Q/TOF-MS-based metabolomic analysis to investigate microbial diversity as well as microbial and metabolite biomarkers in dry-cured ham. In total, detection of 269 different compounds occurred, comprising 49 amino acids, 108 lipids and lipid-like molecules, 76 organic acids and derivatives, and nine nucleic acids, nucleotides, and analogues. The most differential metabolites were amino acids such as carnosine, L-Glutamic acid, L-Norleucine, alpha-linolenic acid, myristoleic acid, ricinoleic acid, L-Histidine, carnosine, and there were significant correlations between them and microorganisms. The amino acid levels in dry-cured hams were significantly higher than in fresh meat control samples, revealing the largest number of the respective metabolite produced during the ripening process. Therefore, they would significantly affected ham’s flavor, as amino acids are the precursors of numerous volatile compounds and play an essential and crucial role in flavor formation of dry-cured ham [46].

Chinese fermented sausages are typical dry-fermented meat products without starter culture [57,58,59]. Recently, Zhou et al. [39] investigated the inhibitory effect of gallic acid on biogenic amines and nitrosamines in Chinese fermented sausages with reduced-nitrite. Using UHPLC-MS/MS-based metabolomic analysis, Zhou et al. [39] identified 719 metabolites, including 285 organic acids and derivatives, 157 lipids and lipid-like molecules, 78 organophosphorus compounds, 54 organic oxygen compounds, 13 organic nitrogen compounds, and other metabolites such as benzenoids, phenylpropanoids and polyketides, alkaloids and derivatives, organosulfur compounds, hydrocarbons, and others. Furthermore, 51 differential metabolites were identified between reduced-nitrite Chinese fermented sausages and control samples. The main metabolites were organic acids and derivatives, and lipids and lipid-like molecules. Research showed, firstly, that gallic acid and nitrites probably have a remarkable impact on lipid metabolism, and secondly, that the use of gallic acid can be a powerful tool in controlling the levels of dimethylamine and trimethylamine in reduced-nitrite Chinese fermented sausages.

Rocchetti et al. [40] also investigated ripened Italian salami produced with 0.5% glucose and mixed starter cultures. Using UHPLC-HRMS-based metabolomic analysis, 1841 metabolites were detected, including amino acids, peptides, glycerolipids, and nucleic acids, highlighting the role of glucose addition in relation to starter culture inoculation.

Liao et al. [43] used LC-MS/MS-based metabolomic analysis to investigate metabolites in Jinhua ham, a traditional Chinese dry-cured ham. Clear metabolic differences were observed between normal and spoiled ham, indicating that spoiled samples could be distinguished from normal ones. According to this study 42 metabolites were identified included 10 amino acid derivatives, 19 peptides, nine organic acids, and four nucleotides. Differential metabolites between normal and spoiled ham were peptides and amino acid derivatives. More specifically, inosine, N-acetyl-Trp, Ile-Lys-Thr-Lys, Lys-Lys-Asn-Lys, N-stearoyl-Val, N-nonanoyl-Gly, Arg-Ile-Ile, acetyl-Leu, Ile-Pro, N-acetyl-Met, 11-oxooctadecanoic acid, N-lactoyl-Tyr, and Asp-Leu were identified as significantly differential metabolites. Notably, the concentration of Ile-Lys-Thr-Lys in spoiled ham was 2.2 times greater than normal ham, being responsible for the higher bitterness of spoiled ham. Additionally, the contents of organic acids (α-licanic acid, 2-hydroxyhexadecanoic acid, and 11-oxooctadecanoic acid) were significantly higher in spoiled ham than normal ham, revealing that it could be the main reason why spoiled hams are extremely sour. The contents of fatty acids (2-hydroxyhexadecanoic acid, 16-hydroxyhexadecanoic acid) were also higher in spoiled ham compared to normal ham, indicating the promotion of fat degradation by spoiled hams. Nevertheless, purine metabolism, pyrimidine metabolism, and protein degradation were the core metabolic pathways in spoiled ham.

Jiang et al. [41] used HS-SPME-GC–MS-based metabolomic analysis to investigate the volatile metabolite profile of fermented dry-cured mutton sausages, focusing on the effect of inoculated fermentation with a mixed culture of *Pediococcus acidilactici* and *Rhizopus oryzae*. In total, 92 volatile metabolites were identified, of which 46 were present in both traditionally fermented and inoculated samples. Furthermore, 12 key volatile metabolites were identified regarding inoculated fermentation, and 5 aldehydes were increased in inoculated samples, such as (E)-2-decenal, (E)-nonenal, 2-undecenal, octanal, and E-2-octenal, thus indicating that aldehydes were the largest abundant group in dry-cured mutton sausages. The aforementioned aldehydes were formed through lipid oxidation and participate in flavor development. High concentration of 1-octel-3-ol and 4 esters were also detected at the end of ripening, including ethyl caproate, ethyl laurate, ethyl palmitate, and ethyl heptanoate. Esters mainly originated from the esterification of acids and alcohols and were correlated with the green, fruity, sweet, and floral odors. In addition to the volatile metabolites, non-volatile metabolites were also investigated using UHPLC-MS/MS-based metabolomic analysis. In total, 779 and 350 non-volatile metabolites were identified in traditional and inoculated fermentation, respectively. Slow metabolomic changes in both traditional and inoculated fermentation took place. A total of 39 and 18 significant different non-volatile metabolites were screened in traditional and inoculated fermentation, respectively, included 20 lipids (12 polyunsaturated fatty acids and phospholipids), eight amino acids and four peptides, eight organic acids, four carnitine, three sugars, and 10 others. Oxidation of phospholipids and lipid intermediates interactions with Maillard reaction resulted in the production of various odor active compounds. Additionally, the level of phospholipids was reduced in inoculated sausages, indicating the increased rate of oxidative lipolysis and, as a result, the acceleration of the ripening process of dry-cured mutton sausages. In addition, the total amino acid content was increased in inoculated sausages, and these eight significantly different amino acids (L-ornithine, L-glutamic acid, tryptophan, proline, valine, D-proline, L-histidine, and L-methionine) improved the sensory properties of sausages [41]. Moreover, in inoculated sausages, the level of sugars was significantly dropped after four days, whereas the content of organic acids increased, thus affecting the flavor. In summary, the addition of starter culture in sausages sped up sugar consumption, decreased the ripening time, and raised the levels of several functional compounds.

## 4. Discussion

Meat products produced by fermentation are a traditional food with specific microorganisms under natural or controlled fermentation conditions, which induces a series of biochemical reactions and physical changes [30]. In addition, these fermented meat products rely on microbial and enzymatic activities for the flavor formation [43,44,45,46]. The use of starter cultures is common, and the main microorganisms include LAB species, micrococci, staphylococci, molds, or yeasts [60]. It is important to note that the addition of starter culture in meat products increases the overall abundance of LAB and decreases the number of spoilage bacteria [41].

Certain LAB strains used in fermented foods may exhibit probiotic potential when their beneficial effects on the host are demonstrated, for example through modulation of the intestinal microbiota [61,62]. Fermented meat products may contain LAB genera such as *Lactobacillus*, *Lactococcus*, *Leuconostoc,* and *Pediococcus*, together with other technologically relevant microorganisms, including coagulase-negative staphylococci, yeasts, and molds [30,60,63]. During fermentation, LAB can produce several metabolites, such as short-chain fatty acids, amines, bacteriocins, exopolysaccharides, and vitamins [28], and they are among the key microorganisms associated with fermented meat products [63]. Moreover, LAB and other starter microorganisms contribute to proteolytic and lipolytic reactions in the food matrix, resulting in breakdown of proteins, fats, and carbohydrates into small molecules [30].

Some of the LAB are highly adapted to meat environments [19] and have also been found to affect the composition of volatile compounds in meat products [20] by affecting the level of metabolites during fermentation [29]. It is worth noting that some of the metabolites produced through fat oxidation, protein hydrolysis, and glycogenolysis interact with each other to form esters, alcohols, and so forth, thus improving the quality of fermented meat products [30]. Thus, a metabolomic approach to LAB can be used in order to monitor the consumption of amino acid and accumulation of organic acids as well as volatile organic compounds [19].

Based on the reviewed data, NMR-based and MS-based metabolomic platforms, together with statistical approaches, have been widely applied to the study of fermented meat products. NMR-based metabolomics is rapid and non-destructive and can provide metabolic fingerprints with minimal sample preparation [23], although it has lower sensitivity and may suffer from spectral overlap [27]. MS-based metabolomics, commonly coupled with chromatographic or electrophoretic separation such as GC, LC, or CE, provides higher sensitivity and broader metabolite coverage and is particularly useful for secondary metabolite analysis [24,25,26]. These analytical platforms, along with statistical approaches and bioinformatics tools, can support the prediction of quality, adulteration, processing effects, and authenticity in meat products [64], as well as the differentiation of naturally fermented and artificially inoculated samples through biomarker discovery and key metabolite identification [22]. Considering this and the complexity of the food matrix, metabolomics alone or in combination with other omic technologies such as genomics, transcriptomics, proteomics and, more recently, peptidomics, metabolomics, and lipidomics, can be used for the holistic characterization of fermented meat products. Thus the mechanisms and affected pathways in meat quality, the differential metabolites and candidate markers in meat products, as well as the new indicators of meat quality, like organoleptic characteristics, freshness, genetic origin, geographic origin, etc. [65,66], becomes apparent. Moreover, as Wang et al. [30] stated, in the future, researchers will use a combination of omic technologies such as metabolomics, metagenomics, proteomics, etc., with sensory analysis of the fermented meat products originating from different regions or areas.

Although metabolomic approaches have improved the characterization of fermented meat products, the available studies still present several limitations. More specifically, MS-based platforms, especially GC-MS, LC-MS, CE-MS, as well as their high-resolution variants, provide high sensitivity and broad metabolite coverage. These platforms are particularly useful for detecting volatile compounds, amino acids, peptides, organic acids, lipids, and other non-volatile metabolites [24,25,26]. In contrast, NMR-based approaches are rapid, reproducible, and require limited sample preparation. They generally show lower sensitivity and may be affected by spectral overlap [23,27]. Therefore, MS- and NMR-based platforms should be regarded as complementary rather than interchangeable approaches.

From the reviewed studies, amino acids, peptides, organic acids, fatty acids, aldehydes, ketones, esters, nucleotides, and biogenic amines emerge as recurrent metabolite groups associated with microbial activity, ripening, flavor formation, and safety-related changes in fermented meat products [22,37,64]. These metabolites are mainly linked to proteolysis, lipolysis, carbohydrate fermentation, amino acid metabolism, and lipid oxidation [30,37]. However, many reported biomarkers remain study-specific, and further validation is required before they can be used as robust indicators across different fermented meat products. Their validation is complicated by differences in starter cultures, meat matrices, fermentation conditions, ripening time, extraction procedures, analytical platforms, and statistical workflows. Therefore, future studies should combine untargeted screening together with targeted validation, standardize sample preparation, and harmonize data processing as well as integration with microbiological and sensory data, in order to strengthen the biological interpretation and comparability of metabolomic findings in fermented meat products.

Microbial communities responsible for the ripening of meat product were investigated [37,47,48,49], giving a microbiota viewpoint of the respective products. In general, it seems that microbial species diversity could lead to various metabolic behaviors that possibly influence the volatile and nonvolatile metabolite profiles of the fermented meat products [37]. Meanwhile, LAB are used to increase the nutrition of food, mitigate the level of harmful microorganism, prolong shelf-life, improve color, flavor, and pH of meat products, etc. [28,32].

Selected LAB strains used as starter cultures can contribute to fermented meat products by producing aroma-related compounds and supporting the standardization of fermentation processes. This, in combination with the continuous development of the food industry, could vastly improve the food safety of fermented meat products as well as their stability. Despite that, the fermentation of most ordinary fermented meat products depends on the role of microorganisms in the native environment, which has been insufficiently researched. Thus, further research is needed on the metabolic pathways and metabolic profiling of LAB and starter cultures in fermented meat products in order to achieve practical implementation in the food industry.

Identification of chemical compounds associated with key meat quality attributes, including spoilage detection, food safety, and authenticity, arises from advances in high-throughput mass spectrometry, as recently reported by [67,68,69]. In beef, exudate-based metabolomic profiling has recently been applied to monitor spoilage development and pathogen contamination [70]. Beef exudate contains a complex mixture of water-soluble compounds, including sarcoplasmic proteins, heme molecules, nucleotides, peptides, free amino acids, soluble enzymes, and hydrophilic vitamins. Through metabolomic profiling, 49 spoilage-associated metabolites were identified in the first experiment, including hypoxanthine, xanthine, trimethylamine, acyl-coenzyme A, nicotinamide adenine dinucleotide (NAD), and adenosine monophosphate (AMP), while 40 metabolites associated with *Salmonella* inoculation, predominantly amino acids and peptides, were identified in the second experiment. Metabolomic approaches applied in meat products have been reported in Table 2.

**Table 2:** Metabolomic approaches applied in meat products. Adapted from Sidira et al. [64] and Wang et al. [29]; Rocchetti et al. [54]; Muhialdin et al. [55]; Mu et al. [34]; Yang et al. [37]; Zhang et al. [71]; Sugimoto et al. [45]; Shi et al. [46]; Belleggia et al. [47]; García-García et al. [56]; Zhu et al. [38]; Zhou et al. [39]; Rocchetti et al. [40]; Liao et al. [43]; Jiang et al. [41]; Setyabrata et al. [68,69]; Abdelhaseib et al. [70]; Fan et al. [72]; Wang et al. [73].

Meat Substrate	Extraction Method	Metabolite Identification Methods	Data Analysis	Results	Reference
Beijing You chicken		HPLC-QTRAP-MS	SPSS 22.0, one-way ANOVA and Ducan’s test, PCA, orthogonal projection to latent structures (OPLS-DA)	In total, 544 metabolites were sorted into 32 categories. L-carnitine, L-methionine and 3-hydroxybutyrate increased with age.	[74]
Chicken, turkey, mixed ground meat for sausages		HPLC-HRMS–Q-Orbitrap	Hierarchical clustering analysis for BWC and VP, one-way ANOVA with Tukey post hoc test, multivariate paired *t*-test.	Irradiation did not cause changes in main food ingredients such as free amino acids, only altered a few metabolic pathways.	[75]
Goose meat	1.0 mL pure methanol (or 70% aqueous methanol) containing 0.1 mg L–1 lidocaine for lipid-solubility metabolites	UPLC-ESI-MS/MS	OPLS-DA, K-means cluster, KEGG	Sorting of 776 metabolites into 16 classes. Increase of carnitine, anserine, nicotinamide riboside with age. Conversely, decrease of hypoxanthine, 2-methylsuccinic acid, and glutaric acid with age.	[76]
Liancheng white duck breast meat and Cherry Valley duck meat	800 mL of icy cold solvent (methanol/acetonitrile1:1, *v*/*v*)	UHPLC-QTOF-MS	SPSS 17.0, one-way ANOVA and Mann–Whitney test, PCA, OPLS-DA	Significant differences between the two breeds; 28 differentiated metabolites were classified. Carbohydrates, amino acids, fatty acids, and eicosanoids were the main ones.	[77]
White and Black Tibetan sheep	1 mL of n-hexane, with 5mL of pure water subsequently added for washing	UPLC-QTOF-MS, NMR for targeted, UHPLC-QTOF-MS/MS for untargeted	PCA	Black Tibetan sheep were superior to the White Tibetan sheep; identification of 49 differential metabolites, including carbohydrates, amino acids and derivatives, fatty acids and derivatives, and other organic compounds.	[78]
Chicken	Solid-phase microextraction (SPME)	UHPLC-Orbitrap MS	PCA, OPLS-DA	Detection of 821 metabolites and division into 16 classes. The amino acids and their metabolites class was the largest (314 metabolites) followed by organic acids and their derivatives (102 metabolites)	[79]
Sour meat	1 mL of tissue extract (75% 9:1 methanol: chloroform, 25% H_2_O)	GC-MS	PCA, OPLS-DA, one-way ANOVA	Identified 68 and 77 differential metabolites in low-salt and traditional sour meat. Level of free amino acids in low-salt sour meat was higher than in traditional sour meat.	[29]
Salami	10 mL of an 80% methanolic solution acidified with 0.1% formic acid using a homogenizer	UHPLC- QTOF-MS	HCA, PCA, OPLS-DA, one-way ANOVA	Identified 111 metabolites. Of these, fatty acyls and glycerophospholipids were the main ones.	[54]
Chicken meat	mixture of CH_3_OH-d4 (0.375 mL) and 0.375 mL KH_2_PO_4_ buffer in D_2_O (pH 6) containing 0.1% TSP as internal standard.	^1^H NMR	one-way ANOVA, Tukey’s test	In total, 25 metabolites were identified.	[55]
Panxian ham	3 mL extraction liquid (V_Methanol_: V_Chloroform_ = 3:1), then 5 mL of L-2-Chlorophenylalanine (1 mg/mL stock in dH_2_O) was added as internal standard followed	GC-TOF-MS	PCA, OPLS-DA, CV-ANOVA, one-way ANOVA, KEGG	In total, 226 metabolites were detected and 31 significantly different metabolites were identified,	[34]
Fuet, spontaneously fermented sausage, and inoculated fermented sausage with commercial starter culture	headspace solid-phase microextraction	UHPLC- QTOF-MS	PCA, PLS-DA, Pearson’s correlation coefficient (PCC)	Many differential metabolites were identified including amino acids, dipeptides, carbohydrates, organic acids, fatty acids, and phosphocholine derivatives	[37]
Dry-cured ham	600 μL of methanol/water (2:1, *v*/*v*)	^1^H-NMR	PCA, OPLS-DA	In total, 28 metabolites were detected including amino acids, peptides, organic acids, nucleic acids and their derivatives, sugars, etc.	[71]
Dry-cured ham	methanol (500 μL) containing (20 μM each) methionine sulfone, D-camphor-10-sulfonic acid, and 2-(N-morpholino) ethanesulfonic acid as internal standards	CE-MS	one-way ANOVA, Bonferroni’s multiple comparison tests, Pearson’s correlation coefficient	Various hydrophilic molecules, such as amino acids (glutamine, cysteine, asparagine, leucine), organic acids, peptides, nucleotides, and their intermediate metabolites were quantified	[45]
Dahe black pig dry-cured ham	SPME	SPME GC-MS	ANOVA, OPLS-DA	In total, 407 volatile compounds were identified during the ripening process. Main volatile compounds formed at 210 days of ripening. Aldehydes and alcohols were the most abundant flavor compound.	[46]
Ciauscolo salami	65 μm PDMS/DVB SPME fiber	SPME GC-MS	one-way ANOVA, PCA, HSD test	In total, 53 volatile substances were identified. The most represented were monoterpene and sesquiterpene including limonene, sabinene, α-pinene, β-pinene, 3-carene, α-thujene, and β- copaene, α-copaene, respectively. Allyl methyl sulphide and diallyl sulphide, together with diallyl disulphide and allyl methyl disulphide, were the major aliphatic sulphur compounds.	[47]
Salchichon dry-fermented sausages	A D_2_O solution (15 μL) containing trimethylsilyl 3-propionic acid sodium salt (TSP, 0.1 mM) was added to the 30 μL disposable Kel-F HR-MAS inserts	^1^H HR-MAS NMR	one-way ANOVA, Bartlett’s test, Duncan’s test, PCA	In total, 177 metabolites were identified, including amino acids, peptides and analogues, carbohydrates, organic acids and derivatives, nucleosides, nucleotides and analogues, fatty acids and miscellaneous, such as Acetone, Crn, Cho, PCho, GPCho, or IMP	[56]
Dry-cured ham	V _methyl butyl ether_: V _methanol_ = 5:1),	LC-Q-TOF-MS	Student’s *t*-test, PCA, OPLS, one-way ANOVA	In total, 269 different compounds were detected, comprising 49 amino acids, 108 lipids and lipid-like molecules, 76 organic acids and derivatives, and nine nucleic acids, nucleotides, and analogues.	[38]
Chinese fermented sausages	QuEChERS method (Quick, Easy, Cheap, Effective, Rugged, and Safe) solid phase extraction	UHPLC-MS/MS	ANOVA, PCA, OPLS-DA	In total, 719 metabolites were identified, including 285 organic acids and derivatives, 157 lipids and lipid-like molecules, 78 organophosphorus compounds, 54 organic oxygen compounds, 13 organic nitrogen compounds, and other metabolites like benzenoids, organic nitrogen compounds, phenylpropanoids and polyketides, alkaloids and derivatives, organosulfur compounds, hydrocarbons, and others.	[39]
Italian Salami, dry-fermented sausages	homogenizer-assisted extraction method	UHPLC-HRMS	HCA, PCA, OPLS-DA	Identified 1841 metabolites, included amino acids, peptides, glycerolipids, and nucleic acids, showing a fundamental role of glucose addition in relation to starter culture inoculation.	[40]
Jinhua ham, Chinese dry-cured ham	Lin et al. method [26] 1 mL of mixed solution (methanol:acetonitrile:water = 2:2:1, *v*/*v*/*v*)	LC-MS/MS	HCA, PCA, PLS-DA, KEGG	In total, 42 metabolites were identified including 10 amino acid derivatives, 19 peptides, nine organic acids, and four nucleotides. Differential metabolites between normal and spoiled ham were peptides and amino acid derivatives. Purine metabolism, pyrimidine metabolism, and protein degradation were the core metabolic pathways in spoiled ham.	[43]
Dry-cured mutton sausages	SPME (7.5 mL of sodium chloride and 15 μL of the standard internal Cyclohexanone)	HS-SPME-GC–MS	ANOVA, PCA, PLS-DA,	In total, 92 volatile metabolites were identified, of which 46 were present in both traditionally fermented and inoculated samples, and 12 key volatile metabolites were identified regarding inoculated fermentation.	[41]
Dry-aged beef loins	Bligh–Dyer extraction protocol	UPLC-MS	Multivariate statistical analysis	Mechanisms involved in flavor generation during dry-aging were elucidated.	[68]
Beef exudate	Bligh–Dyer extraction protocol	LC-MS/MS	Multivariate statistical analysis	Meat exudate profiling was used to determine the impact of postmortem aging on oxidative stability of beef muscles.	[69]
Beef exudate/*Salmonella* contamination	Bligh and Dyer method	LC-MS/MS	Multivariate statistical analysis	Spoilage-associated metabolites and key metabolites associated with *Salmonella* contamination were detected.	[70]
Harbin dry-sausages	headspace solid-phase microextraction (HS-SPME)	LC-MS/MS	Multivariate statistical analysis	Staphylococci contributed to flavor formation through protein hydrolysis and amino acid metabolism.	[72]
Dry-sausages from Northeast China	headspace solid-phase microextraction device	GC-MS	Comparative analysis	Quality and flavor differences between traditional and conventional dry-sausages were characterized.	[73]

Hierarchical Cluster Analysis (HCA), Principal Component Analysis (PCA), Orthogonal Projections to Latent Structures Discriminant Analysis (OPLS-DA), Partial Least Square Discriminant Analysis (PLS-DA), Analysis of variance (ANOVA), Honestly Significant Difference (HSD), Pearson Coefficient Correlation (PCC), Statistical Package for the Social Sciences (SPSS), Kyoto Encyclopedia of Genes and Genomes (KEGG).

Regarding fermented meat products, non-targeted metabolomics has been employed for the detection of non-volatile small molecule metabolites (peptides, free amino acids (FAA), nucleotides, organic acids, etc.) [72,73,80]. In parallel, gas chromatography–mass spectrometry (GC–MS) has been applied for the comprehensive profiling of volatile compounds in fermented meat products [81]. Two strains isolated from dry-sausages, including *S. vitulinus* and *S. equorum* as starter cultures, were shown to affect flavor improvement [82,83]. Fan et al. [72] reported that the amino acid metabolism was a key pathway through which staphylococci contributed to the formation of flavor-related compounds.

In addition, tea polyphenols (TP), particularly their main component gallic acid (GA), have been proposed as natural additives for improving the safety and quality of fermented meat products, as reported by Xia et al. [84]. Metabolomic and metagenomic findings indicated that GA promoted beneficial *Lactococcus garvieae,* while reducing spoilage-associated *Enterococcus faecalis* and *Citrobacter freundii.* Finally, sausages treated with TP showed reduced levels of biogenic amines.

In another study by Liu et al. [85], effects of single and co-fermentation by *Lactiplantibacillus plantarum* and *Debaryomyces hansenii* using different inoculation ratios on physicochemical properties and the non-volatile metabolome of fermented meat were investigated. They showed the effect of the inoculation ratio on acidification, proteolysis, and lipid remodeling during meat fermentation. Amplification of aromatic complexity and reshaping of small-molecule metabolism have been accomplished by the addition of such yeasts, which can act as valuable co-inoculants for diversifying flavor and improving texture [64,86,87]. Non-targeted (LC-HRMS/LC–MS) metabolomics and multivariate tools such as PCA, OPLS-DA, hierarchical clustering, Venn analysis, and KEGG pathway enrichment have been effectively employed to uncover how microbial interactions under different inoculation ratios reshape metabolic networks and promote flavor formation, and reveal associations between amino acid, peptide, lipid and nucleotide metabolism and their associations with sensory and safety indices [88].

The individual and combined inoculation of *Lactobacillus delbrueckii* subsp. lactis N102 and *Latilactobacillus sakei* H1-5 was evaluated in dry-fermented sausages using ^1^H NMR (nuclear magnetic resonance)-based metabolomic profiling [89], showing that both the techno-functional properties and biochemical composition of these products can be enhanced. Similarly, Zhou et al. [90] reported that free amino acids, small peptides, and organic acids were the main metabolites contributing to the taste and flavor development in modern processed hams.

Finally, Li et al. [91] investigated metabolomic changes following single and combined fermentation with *Latilactobacillus sakei* and the non-LAB starter culture *Staphylococcus carnosus* using LC/MS and revealed that the metabolic interactions between *L. sakei* and *S. carnosus* increased the number of functional metabolites in co-fermented sausages.

## 5. Conclusions

Lactic Acid Bacteria (LAB) are used in a wide range of foods. Some species are highly adapted to meat environments and can produce a variety of metabolites, like organic acids, bacteriocin, amino acids, exopolysaccharides, and vitamins, thus improving the flavor and overall acceptance of fermented meat products. Considering this and the complexity of the food matrix, omic technologies like metabolomics, alone or in combination, can be used for the holistic characterization of fermented meat products inoculated with LAB. Mainly two major metabolomic platforms, namely MS-based and NMR-based approaches, have been applied in recent studies. These approaches have helped clarify mechanisms and pathways related to meat quality, identify differential metabolites and candidate biomarkers, and provide new indicators of quality in fermented meat products.

Knowledge of the metabolic activity of LAB and starter cultures in fermented meat products can help industries further develop manufacturing processes and standardize quality parameters. However, in order to understand the overall framework beyond quality variations, extensive and detailed studies are required to incorporate information from different omic technologies. Omic technologies applied to meat products have, with certainty, hopeful prospects for the next years, with key roles in developing the manufacturing processes and standardizing meat quality traits with the use of LAB, helping achieve safety in meat production.

## Data Availability

No new data were created or analyzed in this study. Data sharing is not applicable to this article.
